# Chemical Potential Differences in the Macroscopic Limit from Fluctuations in Small Systems

**DOI:** 10.1021/acs.jcim.0c01367

**Published:** 2021-02-10

**Authors:** Vilde Bråten, Øivind Wilhelmsen, Sondre Kvalvåg Schnell

**Affiliations:** † Department of Materials Science and Engineering, Norwegian University of Science and Technology, NTNU, Trondheim NO-7491, Norway; ‡ SINTEF Energy Research, Trondheim NO-7465, Norway; § Department of Energy and Process Engineering, Norwegian University of Science and Technology, Trondheim NO-7491, Norway; ∥ Department of Materials Science and Engineering, Norwegian University of Science and Technology, NTNU, Trondheim N-7491, Norway

## Abstract

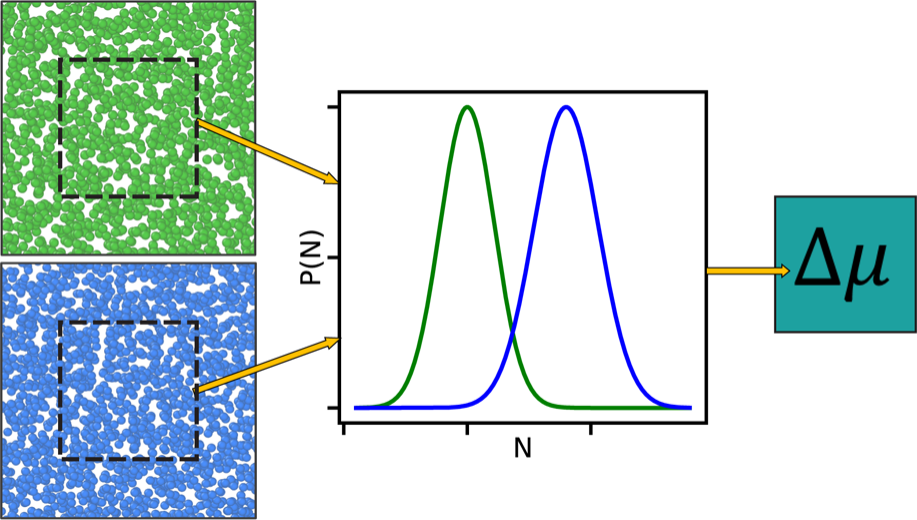

We present a new method for computing chemical potential differences of macroscopic systems by sampling fluctuations in small systems. The small system method, presented by Schnell et al. [Schnell et al., J. Phys. Chem. B, 2011, **115**, 10911], is used to create small embedded systems from molecular dynamics simulations, in which fluctuations of the number of particles are sampled. The sampled fluctuations represent the Boltzmann distributed probability of the number of particles. The overlapping region of two such distributions, sampled from two different systems, is used to compute their chemical potential difference. Since the thermodynamics of small systems is known to deviate from the classical thermodynamic description, the particle distributions will deviate from the macroscopic behavior as well. We show how this can be utilized to calculate the size dependence of chemical potential differences and eventually extract the chemical potential difference in the thermodynamic limit. The macroscopic chemical potential difference is determined with a relative error of 3% in systems containing particles that interact through the truncated and shifted Lennard-Jones potential. In addition to computing chemical potential differences in the macroscopic limit directly from molecular dynamics simulation, the new method provides insights into the size dependency that is introduced to intensive properties in small systems.

## Introduction

Properties available from molecular simulations (MD) can be sorted in two categories: mechanical properties and thermal properties.^1^ The difference between these comes from how they are connected to the partition function. Mechanical properties are related to the derivative of the partition function, while the thermal properties are functions of the partition function itself.^1^ Examples of mechanical properties are therefore the internal energy, pressure, and heat capacity, while examples of thermal properties are Gibbs energy, Helmholtz energy, and chemical potential.

The mechanical properties can be expressed as averages of functions of phase space coordinates and can therefore be calculated directly from the simulation trajectory.^2^ The thermal properties cannot be expressed as such averages. This is because they are related to the complete volume of phase space accessible to the system, which can normally not be sampled in MD.^3^ In order to calculate thermal properties, one must resort to other alternatives than simply analyzing the simulation trajectory. For the Gibbs energy or Helmholtz energy, there are options such as thermodynamic integration^4−8^ or umbrella sampling,^9−11^ while a common method for computation of chemical potential is Widom’s particle insertion method.^2,12,13^ Another route to compute chemical potentials is found in the overlapping distribution methods (ODMs).

The term ODM can be used to describe any method that can extract thermodynamic properties from the overlap between probability distributions of two different systems.^2^ The distributions represent the Boltzmann distribution of the fluctuating properties of the system, which are ensemble-dependent. In the canonical ensemble, where the number of particles, volume, and temperature are constant, the energy will fluctuate. In the isobaric–isothermal ensemble, where the number of particles, pressure, and temperature are fixed, there will be fluctuations in volume and energy. Grand canonical systems, with constant chemical potential, volume, and temperature will have fluctuations in energy and in the number of particles. Naturally, the properties available from the distributions will depend on the ensemble used in the simulation.

For canonical systems, the Helmholtz energy difference is accessible from the overlapping region of two energy distributions. One version of the ODM that utilizes this is the acceptance ratio method presented by Bennett,^14^ who presented strategies for estimating the difference in Helmholtz energy between two canonical systems. Shirts et al.^15^ later showed that it was possible to derive the same expressions from maximum likelihood arguments. Frenkel and Smit^2^ also illustrated how the method can be used to calculate excess chemical potentials. This is achieved by considering two canonical systems where the first contains *N* particles, and the second contains *N* – 1 particles and one ideal gas particle. The Helmholtz energy difference between these systems corresponds to the change in Helmholtz energy in the first system when one of its *N* particles is transformed to an ideal gas particle. Hence, applying the ODM to the energy distributions in these two systems returns the excess chemical potential of the first system.

Even though the most frequent use of the method is calculations of properties in the canonical ensemble,^16,17^ it is not restricted to this. Bennett^14^ showed that it is possible to develop analogous expressions for other ensembles. Recently, Shirts^18^ introduced yet another convenient aspect of the overlapping distributions by using them to determine whether the desired thermodynamic ensemble is properly sampled in the simulations. This ensemble consistency test can be applied to molecular dynamics as well as Monte Carlo (MC) simulations,^19,20^ and it can be used to evaluate simulations performed in the canonical ensemble, isobaric–isothermal ensemble, grand canonical ensemble, or the microcanonical ensemble.

Whether the objective is to use the distributions to calculate thermal properties or to test for ensemble consistency, the starting point is the same: the statistical mechanical connection that exists for every ensemble between its corresponding energy state function and the partition function.

In this work, we will show how the ODM can be used to extract the chemical potential difference of two small grand canonical systems, directly from two MD simulations at different densities. These small systems are generated by placing subsystems at random locations inside the total simulation boxes. The total simulation box can be canonical, microcanonical, or isobaric–isothermal and works as a grand canonical reservoir for the small embedded systems. Hence, fluctuations in the number of particles that arise in the subsystems will not depend on the ensemble of the MD simulation box. These fluctuations represent the Boltzmann distributions of the number of particles in small grand canonical systems. The chemical potential difference between two embedded systems is then available from the overlapping region of two such distributions.

It is also possible to utilize the chemical potential differences in the subsystems to obtain the chemical potential difference for the total simulation boxes, that is, in the macroscopic limit. When investigating these distributions, one must keep in mind that they are calculated in small nonperiodic systems, which means that their thermodynamic properties will deviate from the classical macroscopic behavior. We will therefore use the thermodynamics for small systems developed by Hill.^21,22^ Combined with the proper scaling laws, we are able to obtain the chemical potential difference in the thermodynamic limit.

The idea of using finite-size scaling analysis to obtain thermodynamic properties was explored already in the eighties by Binder’s block analysis method.^23^ In his work, Binder investigated how the probability distributions of the Ising lattice model depend on system size, which in turn was utilized to calculate the magnetic susceptibility in the thermodynamic limit.^23,24^ Binder also extracted values of root-mean-square magnetization and internal energy and explored the possibility of identifying the critical temperature and the critical exponents. This application was later investigated for the two-dimensional Lennard-Jones (LJ) system for both one- and two-phase systems.^25−27^

The method used to create the subsystems in this work is known as the small system method (SSM), developed by Schnell et al.,^28^ and it differs from Binder’s block analysis method in the way the subsystems are created. Binder’s blocks are created as cubic sections in a grid superimposed on the simulation box, while the SSM creates subsystems by placing them at random locations inside the simulation box. One consequence of this difference is that the shape of the subsystems is not restricted to being cubic.^29^

Binder focused largely on critical phenomena and the method’s ability to extract properties in the critical point. Lately, finite-size analysis of subsystems has received more intention in the application to one-phase systems, further from the critical point. It has been used to calculate enthalpies and the thermodynamic factor,^28^ partial molar properties,^30^ and the isothermal compressibility.^29^ For multicomponent systems, the calculation of Kirkwood–Buff integrals^31^ has received much attention due to the connection these integrals have to a number of thermodynamic properties.^32−38^ One of the properties available through the Kirkwood–Buff integrals is the differential chemical potential, which upon numerical integration can provide insights on how chemical potential change with the density of the system.^34,35^ For one-component systems, this can be achieved by investigating the isothermal compressibility.^37,38^ The method we propose in this work does not rely on numerical integration since the chemical potential difference is directly available from the two simulations.

To the best of our knowledge, this is the first time an ODM has been used to extract the properties for small systems. We therefore investigate how well it performs for small grand canonical systems with a MC approach before applying it to the systems generated by the SSM.

For macroscopic systems, the chemical potential is known as an intensive property, meaning that it does not depend on the system size. The newly presented method gives insights on how the chemical potential deviates from this intensive behavior when the system becomes small enough. For calculation of chemical potential differences in macroscopic systems, the method will be particularly useful at high densities, where moves that include insertion and deletion of particles become very inefficient.^1,2,39−41^ Chemical potentials calculated in finite periodic systems are also known to be rather strongly dependent on size.^13,42,43^ This problem is avoided in the method presented here since the macroscopic chemical potential differences are not calculated directly but instead extrapolated to the thermodynamic limit by the use of scaling laws.

## Theory

In the following sections, we present the theoretical background needed for computation of chemical potential differences from fluctuations in small grand canonical systems. The treatment of the thermodynamics of small systems is based on the formalism introduced by Hill.^21^ We will explain how it can be used in combination with scaling laws to obtain properties in the thermodynamic limit. We also explain how the SSM can be used to extract fluctuation in small systems from molecular dynamics simulations. Lastly, we show how the distributions corresponding to these fluctuations can be used to calculate chemical potential differences.

### Thermodynamics for Small Systems

The main difference between small systems and macroscopic systems is usually the surface area-to-volume ratio. Since this ratio is much larger for small systems, the effects of the surface become significant, and the thermodynamic properties can no longer be directly compared to those of macroscopic systems.^21^ This becomes clear by studying the system’s extensive properties, which for small systems will not be proportional to the volume, but higher-order functions of size and shape. The smallness also introduces a size dependence in the system’s intensive properties, which is not present for macroscopic systems. As a result, macroscopic thermodynamic equations cannot be used to describe the properties in small systems.^21,22^

The formalism developed by Hill^21^ provides modified versions of the macroscopic thermodynamic equations that can be applied to small systems. In this derivation, Hill^21^ considered a collection of  small systems that are all equivalent, distinguishable, and independent. The ensemble they make up together can therefore be considered macroscopic, and its differential energy can be expressed as
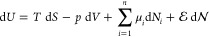
1where *U* is the system’s energy, *T* is the temperature, *S* is the entropy, *p* is the pressure, *V* is the system’s volume, μ_*i*_ is the chemical potential of component *i*, and *N*_*i*_ is the number of particles of component *i* in the system. The property  is called the subdivision potential and is represented by different functions for different ensembles. In the grand canonical ensemble, it is given by . The property *p̂* is known as the integral pressure, which is related to the differential pressure *p* through the following equation
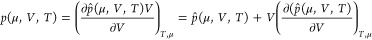
2

The second term in the above equation is only of significant magnitude when the system is small, which means that in the thermodynamic limit, *p̂*(μ,*V*,*T*) = *p*(μ,*V*,*T*).

The two pressures are connected to different types of mechanical work that arise from volume change of the total ensemble, but the mechanisms behind these are not equal. The differential pressure, *p*, is the one associated with the pressure of a macroscopic system. The volume change mechanism connected to *p* must therefore be equal to that of a macroscopic system. This volume change is defined as the change in total volume when changing the volume of all the small system replicas. This represents the work done on the surroundings by the volume change and will be the same whether the systems in the ensemble are small or macroscopic. The work connected to the integral pressure, *p̂*, however, is unique for small systems. In this volume change, the volumes of the small systems are kept constant, while the volume of the total system is changed by adding one replica to the ensemble of small systems. This is done while keeping the entropy and number of particles in the total collection of small systems constant, which means that these properties must be redistributed over all the small systems, including the added replica.

This explains the significance of the different terms in eq 2, but in order to understand its origin, we must look to the connection between the partition function and the energy state function of the system. For a system in the grand canonical ensemble, this is equal to the contribution to the internal energy from the pressure–volume term. In small grand canonical systems, Hill^21^ showed that this relation becomes
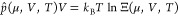
3where *k*_B_ represents the Boltzmann constant and Ξ is the grand canonical partition function. The small system version of the familiar equations for the entropy, pressure, and number of particles in a grand canonical ensemble is
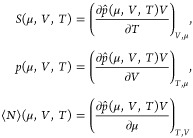
4where the brackets denote average values.

#### Size- and Shape-Dependent Properties

When investigating properties of small systems, it is convenient to use another aspect introduced by Hill.^21^ He argued that a property’s small size contribution can be treated as an excess property, meaning that all dependent properties of a system can be split into a macroscopic contribution and the contribution from finite-size effects. A general property, *A*, can therefore be expressed as
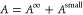
5where *A*^∞^ is the macroscopic contribution and *A*^small^ is the finite-size contribution to *A*. In the thermodynamic limit, *A*^small^ becomes vanishingly small compared to *A*^∞^. Consequently, for macroscopic systems, the property *A* can be regarded as represented by *A*^∞^ only, and macroscopic thermodynamics is applicable. For small systems, *A*^small^ becomes significant, which makes *A* depend on the system’s size and shape, and the macroscopic thermodynamic equations are no longer directly applicable.

In Hill’s^21^ treatment of small systems, only the dependent properties of the system have a finite-size contribution. These are the properties that are not fixed by the system’s ensemble. For a grand canonical system, these are given in eq 4 and can be expressed as
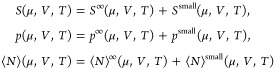
6

A theorem presented by Hadwiger^44^ provides more insights into the meaning of the different terms in the above equations. According to this theorem, any functional of a system that is translationally invariant, additive, and continuous can be written as a sum of four contributions, where one is a constant and the other three are proportional to *V*, *V*^2/3^, and *V*^1/3^, respectively.^45^ The property *A* can therefore be written as
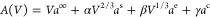
7where α, β, and γ are geometric factors that depend on the shape of the system. In the first term, *a*^∞^ can be understood as the density of *A* in the thermodynamic limit *A*^∞^ = *Va*^∞^, which means that the remaining terms represent *A*^small^. Equations showing the same size and shape dependence have been derived independently and shown to apply to fluids of hard disks,^46^ LJ particles,^47^ and Weeks–Chandler–Anderson particles.^48^

Hadwiger’s theorem can only be used if *A* is extensive, but it is possible to define an alternative equation that applies to intensive properties by dividing eq 7 by the volume
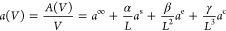
8where we have defined the characteristic length *L* = *V*^1/3^.

### Small System Method

For both macroscopic systems and systems with finite-size effects, knowledge about the fluctuations in energy and number of particles can give access to a large number of thermodynamic properties. The accessibility of these fluctuations depends on the simulation method. Systems with a fluctuating number of particles can normally not be created with MD simulations. In order to simulate grand canonical systems, one can resort to MC simulations, but these are computationally expensive, especially for systems at higher particle densities. The SSM developed by Schnell et al.^28^ offers an alternative way of creating systems with a fluctuating number of particles. In this approach, the grand canonical systems are not simulated directly but instead created by sampling subvolumes from a larger reservoir. The reservoir is typically a large simulation box, which can be simulated using MD or MC. An ensemble average of such a subsampled system is created by placing control volumes of equal size at different locations inside the simulation box.

Some thermodynamic properties have a direct connection to the fluctuations in the number of particles. The origin of these connections is the identity of the second moments of the probability distribution of a grand canonical ensemble
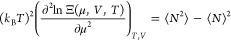
9

These second moments are in turn connected to thermodynamic quantities such as the thermodynamic factor, the isothermal compressibility, the partial enthalpy, and the partial internal energy.^49^ The fluctuations sampled from the subsystem can therefore provide access to a variety of thermodynamic properties. However, it must be kept in mind that the subsampled system cannot be regarded as a representation of the bulk due to the nature of its boundaries. The subsampled system is nonperiodic, which means that it will be affected by a significant contribution from the surface. Since the subsystem represents a small system, its statistics will be different from that of an equivalent system with periodic boundaries. Its thermodynamic properties and the connections between them must therefore be treated with the formalism developed by Hill.^21^

**Figure 1:**
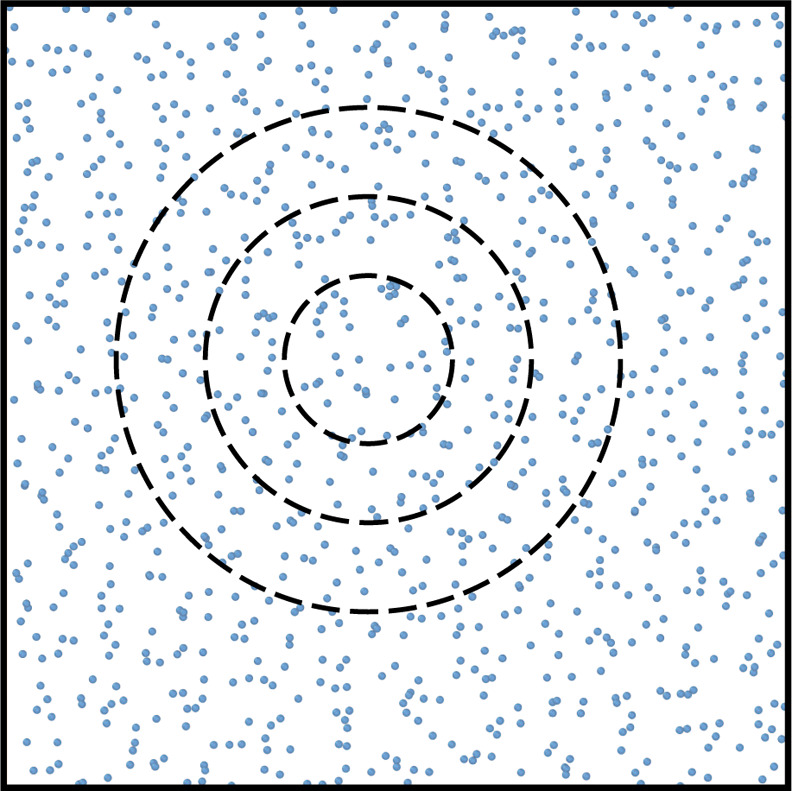
Particle fluctuations are calculated in spherical subsystems inside the total simulation box. The size of the subsystem is gradually increased in order to calculate how the fluctuations change when the size of the system changes.

Since the subsystems are created from control volumes inside a reservoir, we can create systems for a range of different sizes, as illustrated in Figure 1. By systematically changing the size of the subsystem, one can evaluate how its properties change with system size. Combined with the equations provided by Hadwiger,^44^ the different contributions from the different parts of the system’s geometry can be identified (see eq 7). The most popular feature of this method has been its ability to extract the macroscopic contribution, meaning the value of *a*^∞^.^28−30,32,48^ If the main purpose is to extrapolate the values calculated for the subsystems to the thermodynamic limit, the term corresponding to the contribution from the surface usually provides a sufficient description of the size dependency. As a first approximation, eq 8 can therefore be written as
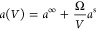
10where we have used that α/*L* = Ω/*V* when Ω is the surface of the system. This expression is particularly useful because it is a straight line if *a*(*V*) is plotted as a function of the surface area-to-volume ratio of the system it was calculated in. The intersection is then equal to the macroscopic contribution.

When using scaling laws to describe size dependence of thermodynamic properties, it is first important to understand which properties will change with size and how this is affected by the type of the system. One important factor to consider for the subsampled systems in the SSM is that they all have the same average particle density. Equation 6 shows that small grand canonical systems will have a finite-size contribution in the number of particles and therefore also to the particle density. However, if this contribution is nonzero in the subsampled systems, it will not appear in their calculated densities. This is because the sampling approach forces the average density of each subvolume to be equal to the reservoir density. In the following, we will explain that if such a size dependency does exist, it will appear in the values of the chemical potentials of the subsystems.

It is important to point out that even though all subsampled systems of different sizes have the same particle density, each individual subsampled system does not have a constant density. The subsampled systems can exchange particles and energy with the surroundings, and they maintain a constant chemical potential due to their connection to the particle reservoir represented by the simulation box. However, the chemical potential does not necessarily remain fixed when the size of the subsystem is changed.

This can be illustrated by considering a general case. We consider two small grand canonical systems with equal average particle density, *n*, and temperature, but different volumes
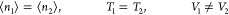
11

Since the systems are grand canonical, their densities can according to eq 5 be written as a sum of the macroscopic contribution and a contribution from the small size
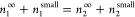
12

From eq 8, we see that *n*^small^ should depend on the system size. Since the two systems considered here have different sizes, their small size contribution are likely to differ, giving *n*_1_^small^ ≠ *n*_2_^small^. According to eq 12, the macroscopic contribution in the two systems will not be equal either, giving *n*_1_^∞^ ≠ *n*_2_^∞^.

The macroscopic particle densities, *n*^∞^, do not depend on size, but they depend on the chemical potential and temperature. Since the temperature is the same in the two systems, a difference in *n*^∞^ must arise from a difference in chemical potential. This means that since *n*_1_^∞^ and *n*_2_^∞^ are different, the two differently sized systems considered here must also have different chemical potentials, that is, μ_1_ ≠ μ_2_. Equation 12 is therefore more correctly expressed as
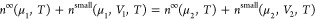
13

This shows that keeping the particle density equal in all the differently sized subvolumes imposes a difference in their chemical potentials.

### ODMs for Small Systems

We will now show how combining fluctuations calculated from two independent systems can be used to extract thermodynamic properties.

The fluctuations in the number of particles represent the Boltzmann distributed probability of finding a certain number of particles in the system. In a grand canonical system, this is given by
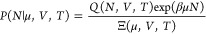
14where β = 1/*k*_B_*T* and *Q* represents the canonical partition function.

This distribution is unique for a given set of chemical potential, volume, and temperature. This means that if one of these is changed, the total distribution will change. This feature is utilized by a number of different methods, which all can be placed in the category of ODMs.^2,14−18^ Common for all of these is that they extract thermodynamic properties from the overlapping region of two distributions sampled from two different states. In this region, the ratio of the two probability distributions gives access to thermodynamic properties through the connection between their respective partition functions and their corresponding energy state function.

Following the procedure of Shirts,^18^ we derive an expression for the ratio of two probability distributions of the number of particles, corresponding to two different grand canonical systems. Moving forward, it must be kept in mind that the goal is to derive a method that can be applied to small systems. This means that we must use equations that take the small system effects into account. Hill’s^21^ equations are convenient because they are valid for small systems and for macroscopic systems. This formalism does not require a separate set of equations in the treatment of small systems since all of Hill’s equations reduce to the corresponding macroscopic identities when the systems become large enough.

Starting from eq 14, we see that *Q* is a function of *N* but not of μ. This means that it is possible to perform two simulations at different chemical potentials but otherwise identical parameters (meaning *T* and *V*) and calculate the ratio of their probability distributions as
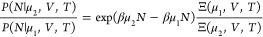
15where the canonical partition function cancels because it has no direct dependence on μ. Taking the natural logarithm of this equation and inserting eq 3 gives
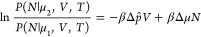
16

This expression is in the form of a straight line, α_0_ + α_1_*N*, if the logarithm of the ratio of the probability distributions is plotted as a function of the number of particles. The values of Δμ = μ_2_ – μ_1_ and Δ*p̂* = *p̂*_2_ – *p̂*_1_ are then readily available since the slope of this line is α_1_ = βΔμ, while the intersection with the *y*-axis represents α_0_ = −βΔ*p̂V*.

The calculation of the distributions is straightforward since the only required information is the number of particles in the systems throughout the simulations. The probabilities can also easily be visualized by binning the particle numbers in histograms. One can even calculate the ratio of the probability distributions directly from the histograms in order to visually inspect that it forms a straight line. Another alternative which is more robust is to use the maximum likelihood approach.^15^ Using this method, the slope can be found from the maximum likelihood expressions for the ratio of the probability distributions. For the grand canonical ensemble, the maximum likelihood expression becomes
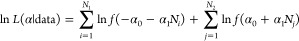
17where *f*(*x*) is the Fermi function *f*(*x*) = [1 – exp(−*x*)]^−1^. The expression only has one maximum, which means that it will always converge, and it can be solved by any standard technique for multidimensional optimization.^18^

The equations presented above can be used to calculate the difference in pressure and chemical potential for two grand canonical systems with different chemical potentials but identical volume and temperature. The subsystems generated by the SSM are examples of such systems. It is possible to investigate the size dependence of Δμ and Δ*p̂*by sampling subsystems over the same size range in two reservoirs at different chemical potentials. In addition, eq 10 can be used to identify the values in the macroscopic limit as Δ*p̂*^∞^ = Δ*p*^∞^ and Δμ^∞^.

## Simulation Details

All systems considered in this work consist of LJ particles that interact via the truncated and shifted potential, with the cutoff radius at 2.5. Unless otherwise specified, all values are presented in reduced units. The critical point of the truncated and shifted LJ system is at *T* = 1.086, *p* = 0.101, and ρ = 0.319.^50^ In all simulations, a temperature of *T* = 1.5 is used.

Four types of systems are investigated:

1. Small grand canonical system with nonperiodic, hard walls. This is simulated using an in-house MC code.
2. Small grand canonical systems with nonperiodic walls, interacting with the particles according to the LJ potential. This is simulated using and in-house MC code.
3. Grand canonical systems with periodic boundary conditions (PBCs). This is simulated using and in-house MC code.
4. Small subsampled systems generated from a large reservoir simulated using the MD code LAMMPS.^51^

The first three system types are used to investigate how well the ODM performs for small systems, compared to systems with periodic boundaries. For these simulations, we use an in-house MC code, with input chemical potentials μ = 0.33, μ = 0.73, and μ = 1.2. For systems of type 3, periodic boundaries are used to remove finite-size effects in order to obtain the system’s bulk properties. For the small systems, type 1 and type 2, the boundaries are treated in a way that introduces a significant contribution from finite-size effects. This is achieved by two different approaches. Systems of type 1 have hard walls, which means that there is no explicit interaction between the particles and the wall, but MC moves that attempt to move a particle outside the simulation box are rejected. Systems of type 2 have a LJ potential with a cutoff distance equal to 1 on the boundaries. This means that any particle within a distance *r*_c_^wall^ = 1 from one of the boundaries interacts with the wall according to the LJ potential. All grand canonical MC systems are cubic, and five different sizes are considered for the small systems, *L* = 5, *L* = 6, *L* = 7, *L* = 8, and *L* = 9, while the periodic system has a size of *L* = 9.

The fourth system type is used in the combination of the SSM and ODM to calculate how the chemical potential difference changes with the system size. The SSM reservoirs are created from molecular dynamics simulations in the *NVT* ensemble using the open source code LAMMPS.^51^ The system’s configuration is stored from the trajectory every 50 time steps from a simulation with a total of one million time steps. For each configuration, 100 randomly positioned points are used to position the center of the small subsystems, giving a total of 2 × 10^6^ samples for each small system volume.

The subvolumes investigated are either spherical or cubic and centered at randomly chosen points, *p*_c_ = (*x*_c_, *y*_c_, *z*_c_). All particles with position *p*_p_ = (*x*_p_, *y*_p_, *z*_p_) satisfying (*x*_p_ – *x*_c_)^2^ + (*y*_p_ – *y*_c_)^2^ + (*z*_p_ – *z*_c_)^2^ ≤ *R*^2^ are placed inside the sphere of radius *R*. For the cubic system, the conditions of the particles’ position become (*x*_p_ – *x*_c_) ≤ *L*/2, (*y*_p_ – *y*_c_) ≤ *L*/2, and (*z*_p_ – *z*_c_) ≤ *L*/2. All particles satisfying these three conditions are placed inside the cubic small system of box length *L*. We investigate 200 differently sized small systems with sizes increasing linearly with the reciprocal radius or reciprocal box length.

One of the conditions for the SSM to work properly is that the investigated state is sufficiently far from the critical point. This is because fluctuations become very long ranging close to the critical point and can therefore not be used to calculate properties accurately.^52^ The simulations are therefore carried out at a temperature of *T* = 1.5.

A second condition for the SSM to give reliable results is that the differently sized subvolumes display a definitive linear region as a function of inverse system size. This means that the simulation box used as the reservoir must be large enough to sample systems in this region. We therefore use cubic simulation boxes containing 27,000 particles at three different number densities, ρ = 0.70, ρ = 0.72, and ρ = 0.74. For these three densities, the macroscopic chemical potential is calculated with the Widom^12^ particle insertion method using an in-house MC code and cross-checked with the TREND equation of state (EOS) provided by Thol et al.^50^

## Results

The purpose of the first section of the results is to investigate the performance of the ODM in small systems compared to how well it performs in a periodic system. Two small grand canonical systems with the same size and temperature but different chemical potentials will have different size effects. We shall investigate whether this will influence the results from the ODM. This section also elaborates on how the density should be calculated in small systems.

The second section contains a description on how the SSM is combined with the ODM to compute the size dependence of chemical potential differences. This includes a guide on how to choose the sizes of the subsampled systems in order to achieve an accurate extrapolation to the thermodynamic limit.

### ODM for Grand Canonical MC Systems

In order to evaluate how well the combination of the SSM and ODM works, we must first evaluate how well the ODM works for small and large systems. This is performed by using the ODM to calculate the chemical potential difference in different types of grand canonical MC systems. The difference between the system types is determined by the way its boundaries are treated. The values of Δμ are calculated by using the maximum likelihood version of the ODM, meaning that eq 17 is applied to the particle numbers calculated from two simulations of grand canonical MC systems. Since μ is one of the input values of a grand canonical MC simulation, the true value of Δμ corresponds to the difference between these two input values.

**Figure 2:**
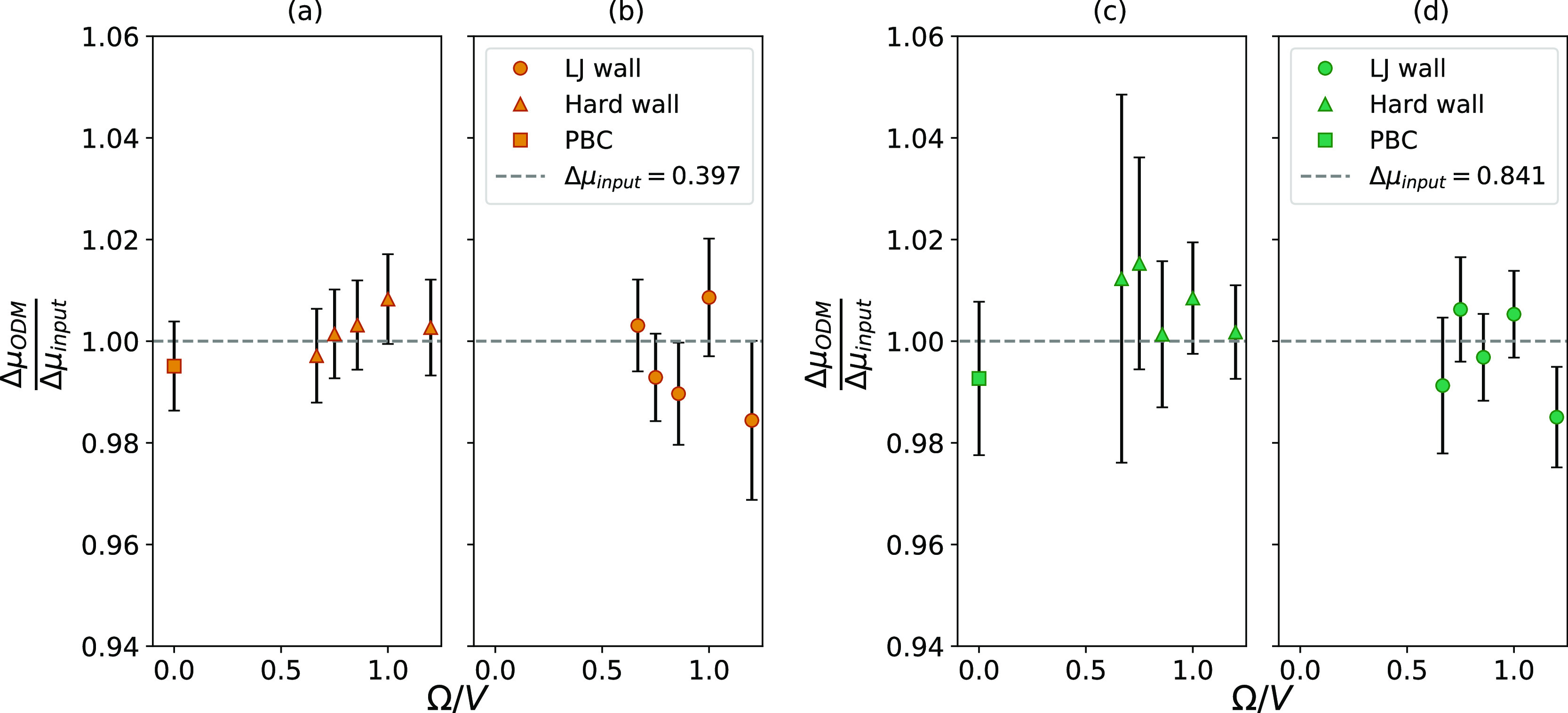
Chemical potential difference calculated from the ODM relative to the input value for different system sizes. Ω/*V* corresponds to the surface area-to-volume ratio. The input value of Δμ is represented by the dashed line, while the symbols show the results from the ODM. The circles represent systems with LJ potential on the boundaries, while the triangles represent systems enclosed by a hard wall. The squares represent PBCs, which gives the value in the macroscopic limit. (a,b) corresponds to the input Δμ = 0.397, while (c,d) corresponds to Δμ = 0.841. All error bars denote two standard deviations.

From the three absolute values of μ, two values of Δμ are calculated: between the two states with the lowest values of μ and between the lowest and the highest values of μ. The standard deviations are computed from 500 bootstrap samples of the total data set. Figure 2 shows the results from applying the ODM to the system with PBC and to the two types of small systems. Figure 2a,b corresponds to Δμ = 0.397, while Figure 2c,d corresponds to Δμ = 0.841. The results are shown as a function of the surface area-to-volume ratio, which is proportional to the inverse system size. This means that the largest systems are found to the left in the figures. The results from the periodic systems represent the macroscopic values and are therefore placed at Ω/*V* = 0. The PBC values are only shown in Figure 2a,c since Δμ in both the hard wall system and the LJ wall system approaches the same value in the macroscopic limit.

The values of Δμ calculated from the ODM show no size dependence for any of the systems considered, and the relative error is below 2% for all systems.

A common criterion for evaluating the accuracy of a method is that its mean value should be within two standard deviations of the true value. Nearly all of the mean values are less than two standard deviations from the true input value; the only exception is the smallest LJ wall system for Δμ = 0.841 (Figure 2d). Since this evaluation is very much dependent on the magnitude of the standard deviations, we investigate the origin of their varying magnitudes.

In the following, we shall see that the magnitude of the standard deviations can mainly be attributed to the choice of the two states investigated. Shirts^18^ pointed out that if the states are far apart, the overlapping region will be small and there will be too few sampled data points, while if the states are too close, the same distribution is essentially sampled from both systems.

The distance between two sampled distributions is determined by the system’s particle densities, which can be size-dependent. The consequence is that this distance for small systems is likely to differ from that of the macroscopic systems. The actual width of the distributions is also likely to change with both size and density. At high densities (ρ ≈ 0.7), small systems usually have larger relative fluctuations than their corresponding macroscopic system.^28,32,48^

**Figure 3:**
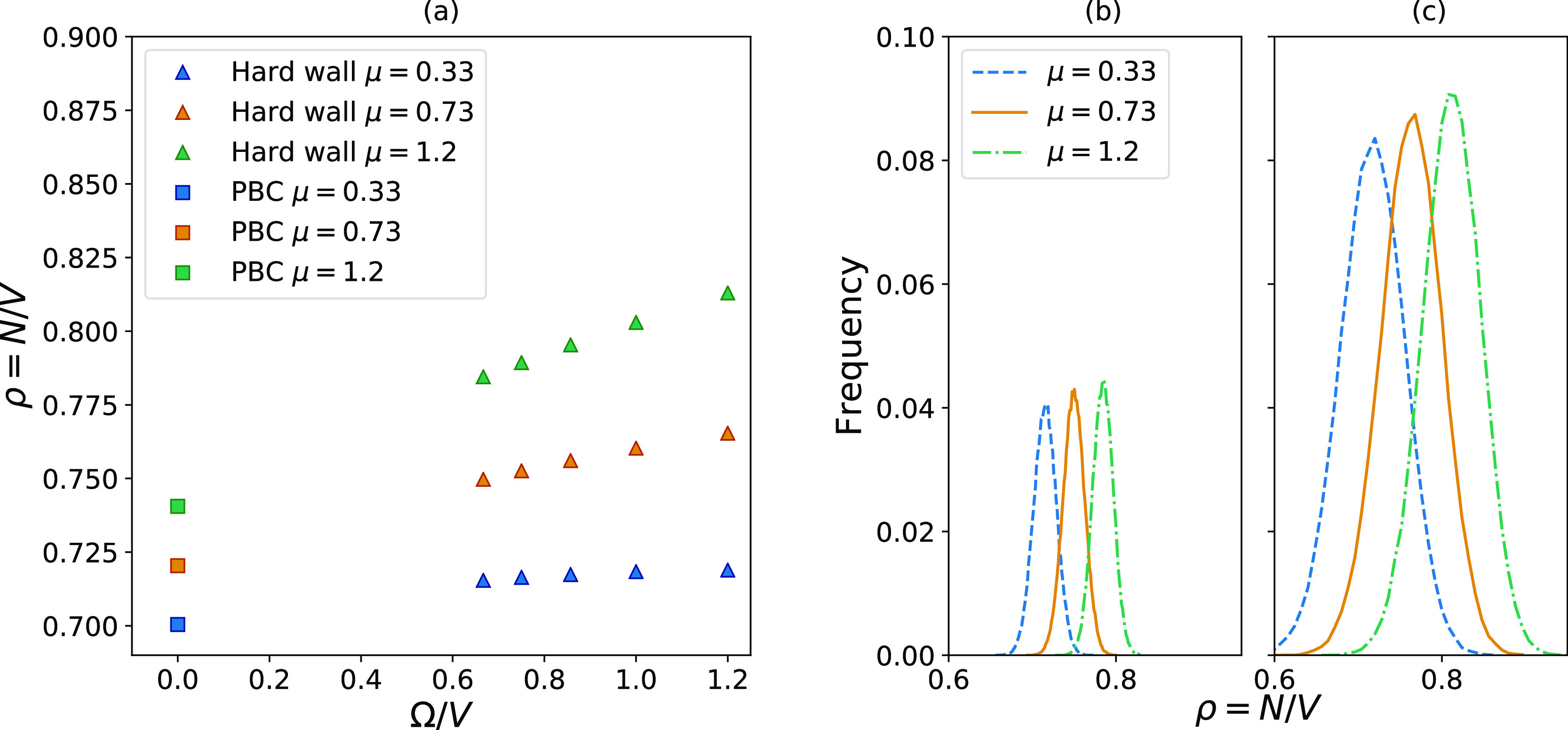
(a) shows how the density in a system enclosed by a hard wall changes with the surface-to-volume ratio when the chemical potential and temperature is kept constant. The triangles represent the hard wall systems, while the squares represent PBCs, which gives the value in the macroscopic limit. (b) shows distributions in density for the largest system, *L* = 9, while (c) shows the one for the smallest system, *L* = 5.

**Figure 4:**
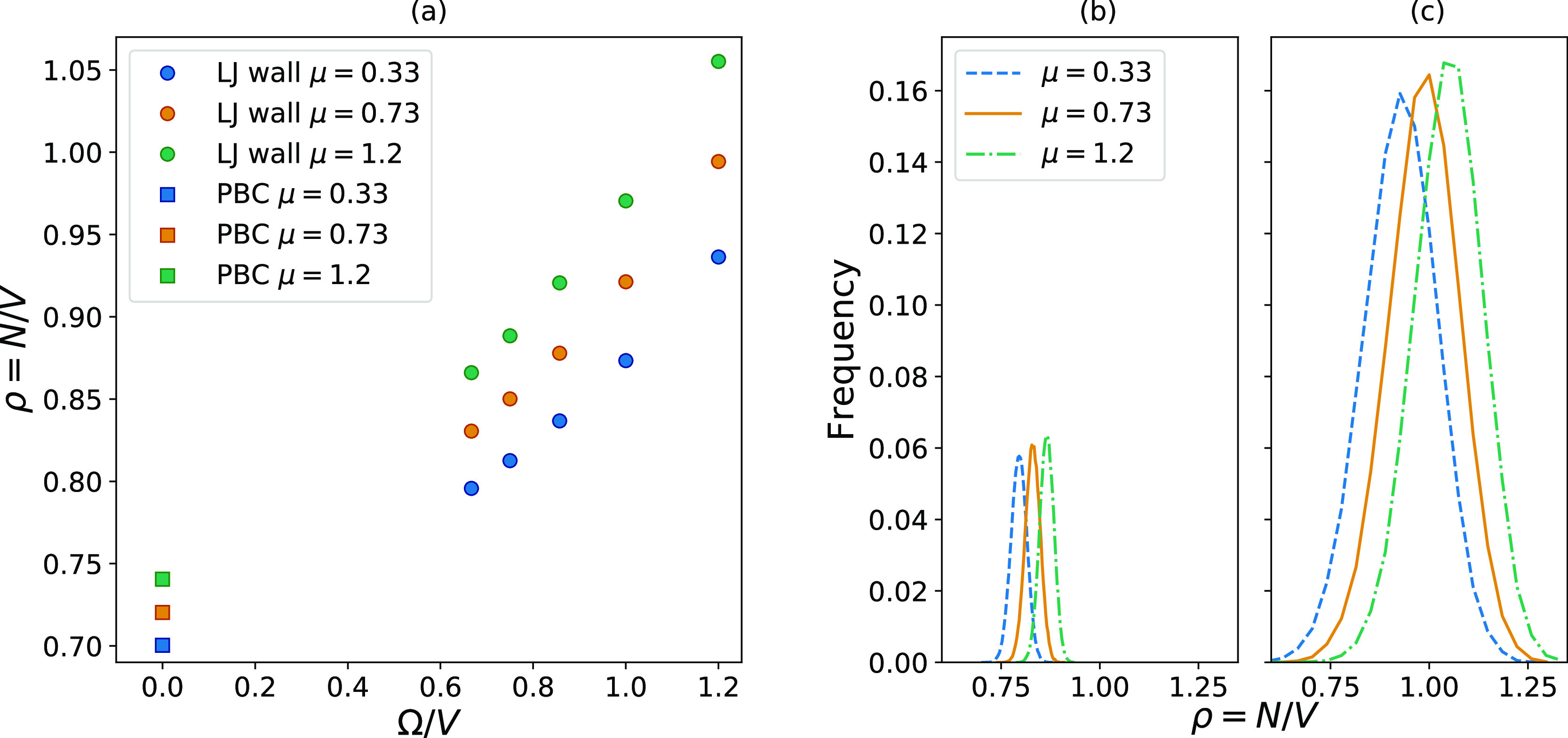
(a) shows how the density in a system enclosed by a wall with a LJ potential changes with the surface-to-volume ratio when the chemical potential and temperature are kept constant. The circles represent the LJ wall systems, while the squares represent PBCs, which gives the value in the macroscopic limit. (b) shows distributions in density for the largest system, *L* = 9, while (c) shows the one for the smallest system, *L* = 5.

Figures 3 and 4 show that both these effects are present for both types of small systems considered here. Figures 3a and 4a show that the density is size-dependent and that the size dependence varies with the value of μ since higher values of μ give steeper slopes. The result is that the distance between the distributions increases when the systems become smaller. The width of the distributions also changes with the system size. Figures 3b and 4b show the density distributions for the largest systems considered, with a size of *L* = 9. Figures 3c and 4c show the density distributions for the smallest systems considered, with a size of *L* = 5. Both types of small systems show wider distributions for smaller systems.

The fact that both the mean value of the densities and the width of the distributions change with size can explain the variation in magnitude of the standard deviations seen in Figure 2. The largest hard wall system shown in Figure 2c is taken as an example. The distributions corresponding to this system are shown in Figure 3b, where the curves corresponding to the highest and lowest values of μ have almost no overlap. This means that there is very little data to calculate Δμ from, which gives this value of Δμ the largest standard deviation in Figure 2c. When the system becomes smaller, as shown in Figure 3c, the distance between the peaks becomes larger, but at the same time, the distributions become wider. This increases the overlap, which reduces the magnitude of the standard deviations.

We conclude that different factors contribute to the magnitude of the standard deviations in different ways and sometimes cancel. Fortunately, the same factors have no significant effect on the mean values since they all have a relative error below 2%. For the systems considered here, the values of Δμ calculated from the ODM are equally reliable for systems with finite-size effects and for systems with periodic boundaries.

#### Calculation of the Density in Small Systems with a Wall Potential

When calculating properties that include the system’s volume, it is common to use the full volume of the simulation box. For a system with periodic boundaries, this is unproblematic. However, if the system is small and has a wall potential, this choice might introduce errors. The reason for this is that the simulation volume is not always equal to the volume available to the center of masses of the particles. This effect has been discussed in a paper by Reiss and Reguera,^53^ where they investigate how neglecting the difference between these volumes can lead to errors in pressures calculated by the virial.

They present results from a simple system consisting of hard spheres with radius ξ inside a spherical simulation volume. The particles interact with the wall such that the center of mass of each particle will never be closer to the wall than a distance equal to the particles’ radius ξ. As a result, the movement of the particles is restricted to a smaller volume than the total simulation volume. Since the virial refers to the volume available to the center of masses of the particles, it results in incorrectly computed pressures when the total simulation volume is used.

The same principles apply when the density of a system is calculated. To get a proper representation of the density experienced by the particles, the volume available to their center of masses should be used. For the hard wall system considered in this work, no correction is needed since the particles do not actually interact with the wall. The particles are allowed to move around in the total simulation volume, but every MC move that attempts to move the center of mass of a particle outside the simulation box is rejected.

For the systems with the LJ wall, however, such a correction must be incorporated. For the case considered by Reiss and Reguera,^53^ finding the volume available to the particles’ center of mass is a trivial task. The radius of the spherical simulation box is simply reduced by ξ. The equivalent distance in the system considered here is the collision radius σ^wall-particle^ between the LJ particles and the LJ wall. This distance is not as rigid as the radius of a hard sphere, but it still gives an indicator of the density experienced by the particles. The densities presented in Figure 4a are therefore calculated by using the corrected box length *L*_corr_ = *L* – 2σ^wall-particle^. They are still plotted as a function of the size of the total simulation box volume, meaning that Ω/*V* is calculated from the noncorrected *L*. Since the effect a system’s wall potential has on its properties is not the main topic of this work, the density calculations will not be discussed further.

### Combining the ODM and SSM

When choosing which system sizes to investigate with the SSM, the most important criterion is that the fluctuations in the subvolumes must represent grand canonical fluctuations. For certain subvolume sizes, this criterion has previously been confirmed by comparing the fluctuations sampled in a closed simulation box to ones computed in subvolumes in true grand canonical reservoirs.^47,48^ In this region, the properties must display a clear linear behavior as a function of inverse system size. This means that the total simulation box must be large enough to act as a grand canonical reservoir for the relevant system sizes. Knowing exactly when the reservoir is large enough is not always a trivial task since this can vary for different types of systems. After calculating the properties, it can also be challenging to identify the linear region. Before evaluating the results of the SSM combined with the ODM, we will therefore present a few tools that can be used to identify the linear region for properties calculated from fluctuations in systems sampled using the SSM. All reported relative errors in the following sections are calculated with respect to the Thol et al.^50^ EOS, unless otherwise specified.

#### How to Identify the Linear Region

Properties in the SSM are calculated from the fluctuations in the number of particles and sometimes also fluctuations in energy.^30^ In this work, we will only consider properties calculated from fluctuations in the number of particles.

When the particle fluctuations are affected by size, the properties calculated from these fluctuations share that size dependence. This means that if it is possible to identify the system sizes that display a linear region for the particle fluctuations, other properties are expected to behave linearly within the same region. The fluctuation in the number of particles is represented by the property
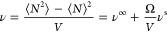
18

This scaling law only describes small size contributions proportional to the surface area. This means that size effects originating from other parts of the system’s geometry, such as curvature, edges, and corners, are not described. System sizes that have a significant contribution from one of these small size effects should therefore not be included in the extrapolation.

In the other end of the size scale, the fluctuations in the largest subvolumes will be affected by the limited size of the reservoir. A density change in a small subsystem cannot happen without a corresponding density change in the reservoir.^29^ This means that as long as the reservoir is closed, it will not act as a proper grand canonical reservoir for the largest subvolumes.^46−48^

In order to identify the linear region, two questions must be answered: (1) What is the smallest volume that is not affected by other finite-size effects than surface area? (2) What is the largest volume that is not affected by the finite size of the reservoir? All volumes in between these limits should be used for extrapolation in combination with scaling laws such as eq 10.

**Figure 5:**
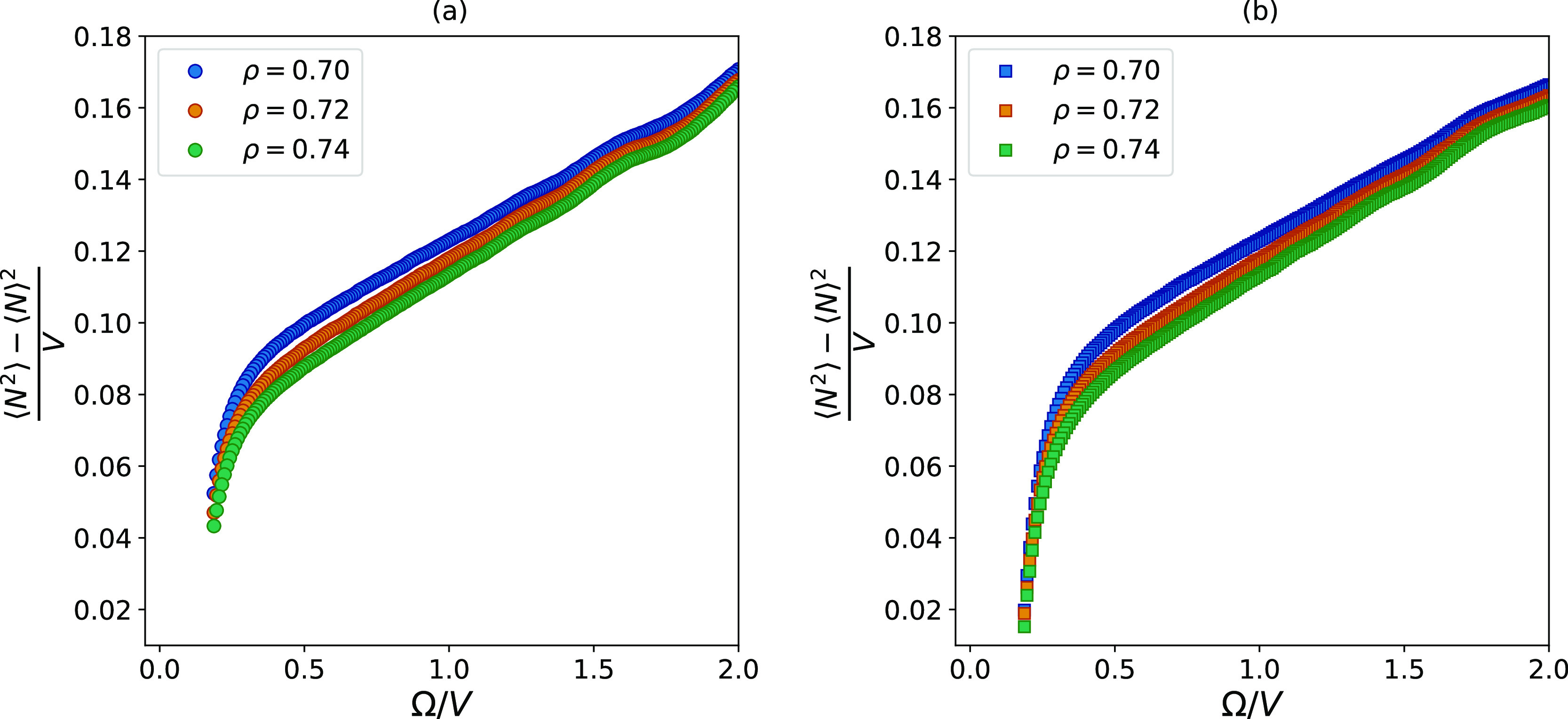
How the value of ν given by eq 18 changes with the surface-to-volume ratio of the subsystem it was calculated in. For the three different curves, the density is constant, equal to that of the reservoir. (a) shows the fluctuations calculated in spherical subvolumes, while (b) shows the fluctuations calculated in cubic subvolumes.

Now, we will show how these questions can be answered for the particle fluctuations calculated in subsystems embedded in simulation boxes at the three different densities considered here ρ = 0.70, ρ = 0.72, and ρ = 0.74. Figure 5 shows the property ν, given by eq 18, as a function of the surface-to-volume ratio.

In order to answer the first of the two questions introduced above, one must look for signs that other finite-size effects than those proportional to the surface area are present.

In Figure 5, the smallest volumes are found at the right side at the highest values of Ω/*V*. In this region, we observe that the fluctuations display a wavy behavior, which indicates the influence by higher-order terms,^48^ as shown in eq 8. Exactly where the wavy region begins depends on the density of the system. This is because higher densities normally cause larger size effects due to a larger number of the particles closer to the surface.^46^

When the aim is to combine the fluctuations in the ODM, a common limit for the linear region should be chosen for the three densities, and it should be based on the behavior of the system with the highest density since this is most influenced by the higher-order terms. The wavy region for the highest density disappears around Ω/*V* = 1.0 for both spherical and cubic subvolumes, which means that subvolumes with a size corresponding to values of Ω/*V* > 1.0 should not be included in the extrapolation.

The other end of the linear region, representing the largest subvolumes, can sometimes be more challenging to determine visually. This is because the impact of the closed reservoir is gradually introduced as the systems become larger. Figure 5 shows that the values of the fluctuations all approach zero in this limit because the reservoir is not able to create large enough fluctuations. Visual inspection alone does not give a clear answer to which value of Ω/*V* the fluctuations start to approach zero. However, it seems to appear at smaller volumes for the system with the lowest density since the fluctuations are larger here, which might be more challenging for the closed simulation box to satisfy.

Since this limit behaves differently for different types of systems and is less apparent than the wavy region, it is helpful to take advantage of some extra tools to identify it. Some general guidelines for identifying this limit have previously been proposed by Cortes-Huerto et al.^35^ and Rovere et al.^27^ These limits are based on the size of the subvolumes relative to the volume of the simulation box, *V*_0_, and correspond to (*V*/*V*_0_)^1/3^ = 0.3 and (*V*/*V*_0_)^1/3^ = 0.25, respectively. The most conservative subvolume sizes identified by these suggested limits are based on the system with the highest density (ρ = 0.74) since this is the one with the smallest simulation box. For spherical subvolumes, the above limits translate to Ω/*V* ≈ 0.49 and Ω/*V* ≈ 0.58, and for cubic subvolumes, they correspond to Ω/*V* ≈ 0.60 and Ω/*V* ≈ 0.72.

We note that these generalized guidelines do not properly represent the situation investigated here since they allow for larger subvolumes when the simulation box is larger. As explained above, Figure 5 shows that the fluctuations in the largest simulation box are the ones most affected by its finite size. In addition, the suggested limits are based on the behavior of fluctuations in cubic subvolumes. Since it has previously been shown that the location of the linear region is dependent on the shape of the subvolumes,^29,48^ it is possible that the predicted limit for a spherical subsystem might not reflect the true location of the linear region. In the following, we therefore investigate two additional tools that can be used directly for the systems under investigation.

One simple option is to fit a straight line to the data points and evaluate at which point the residual, meaning the difference between the fitted line and the actual data points, starts to deviate. The coefficient of determination, also known as *R*^2^, provides an indicator of how well a model describes the data points.^54^ If there is complete overlap between the model and the data points, the value of *R*^2^ is equal to 1, while if *R*^2^ is closer to 0, the data points are uncorrelated and cannot be described by the model.

**Figure 6:**
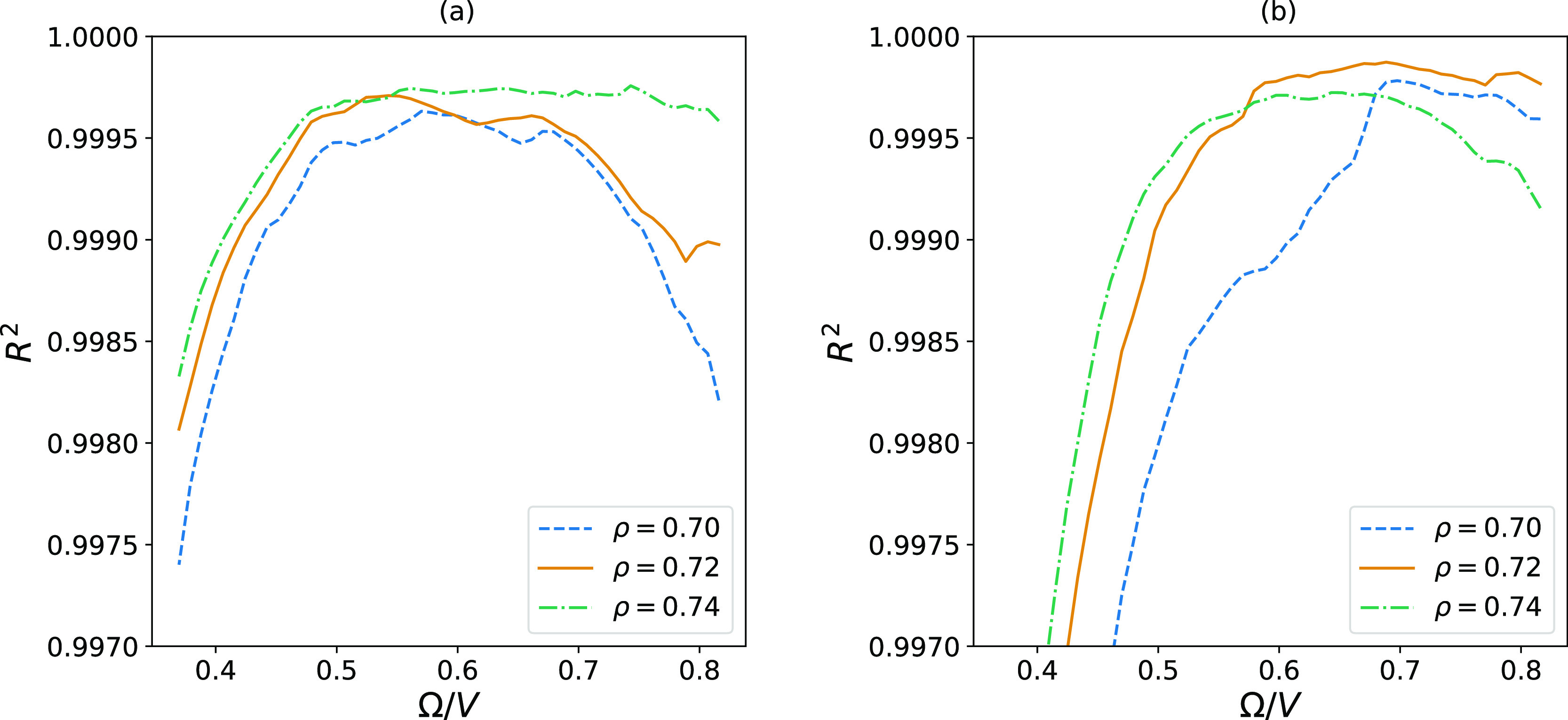
Coefficient of determination, *R*^2^, calculated from a fitted straight line to the data points shown in Figure 5. The values are shown as a function of the surface-to-volume ratio, which means that the value with most included data points is found to the left. The first value of *R*^2^ is calculated based on a linear fit of the data points between Ω/*V* = 1.0 and Ω/*V* = 0.8 and then gradually updated as more data points are included in the linear fit. (a) shows the case for spherical subvolumes, while (b) represents cubic subvolumes.

Figure 6 shows how the coefficient of determination changes as a function of data points included in the linear fit. The values of *R*^2^ are shown as functions of the surface area-to-volume ratio, which means that the number of data points included in the linear fit increases to the left in the figure. The first values of *R*^2^ (furthest to the right in Figure 6) are calculated based on a linear fit of the data points between Ω/*V* = 1.0 and Ω/*V* = 0.8 in Figure 5. The remaining data points are then included in the fitting one by one, and *R*^2^ is recalculated based on the new linear fit.

We observe that when more data points are included in the fitting, the values of *R*^2^ initially approach 1. However, at one point, the *R*^2^-values start to deviate. This means that we have reached the regime where the simulation box no longer functions as a proper grand canonical reservoir. For systems with the lowest density, this deviating behavior is observed to start for smaller subvolumes, where it is more challenging for reservoirs to satisfy the fluctuations in their subvolumes. We therefore choose the limit based on the curve for the lowest density, which starts to deviate from 1 around Ω/*V* = 0.57 for the spherical subvolumes and for Ω/*V* = 0.72 for cubic subvolumes.

A more advanced alternative is to compare the results found by linear fitting to values extracted by an equation that explicitly includes the effect of the finite size of the simulation box. The possibility of including effect in scaling equations has been explored to a large extent in the literature.^35,37,46,48,55,56^ One such equation was proposed by Strøm et al.^29^ and is given by
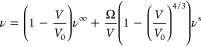
19

This equation is derived by assuming that ν = 0 for *V*/*V*_0_ = 1, which only is valid for subvolumes of the same shape as the reservoir. We apply eq 19 to the fluctuations calculated in the cubic subvolumes with size 0.4 < Ω/*V* < 1.0. The largest subvolumes (Ω/*V* < 0.4) are not included in the fitting of eq 19 since this resulted decreased accuracy in the values of ν^∞^. We believe that this can be explained by two factors. The first is that the fluctuations in larger systems usually converge more slowly than their smaller counterparts.^25,26^ The second is that fluctuations in subvolumes of a size comparable to the total simulation box can become influenced by its periodic boundaries.^47^

The relative errors of ν^∞^ extracted from eq 19 are below 1% for all densities investigated. This means that the values of ν^∞^ obtained from this fit can be compared with the values extracted from the linear fitting and thereby work as a quality check for the limits identified by analyzing *R*^2^. The ν^∞^-value obtained from eq 19 differs by 4% from the value obtained from linear fitting to spherical volumes in the range 0.57 < Ω/*V* < 1.0 and by 6% from linear fit to cubic subvolumes in the range 0.72 < Ω/*V* < 1.0. It is also possible to systematically vary the limits until we reach the minimum difference between the values extracted from the two types of curve fitting. By changing the lower limit of Ω/*V* to 0.60 for spherical subvolumes and to 0.78 for cubic subvolumes, this difference is reduced by 1%-point. Changing the limits beyond these values does not decrease the difference further.

We note that an equation similar to eq 19, which also takes the finite size of the simulation box into account, has been developed by Cortes-Huerto et al.^35^ and that this equation should work for the same purpose as described above.

**Figure 7:**
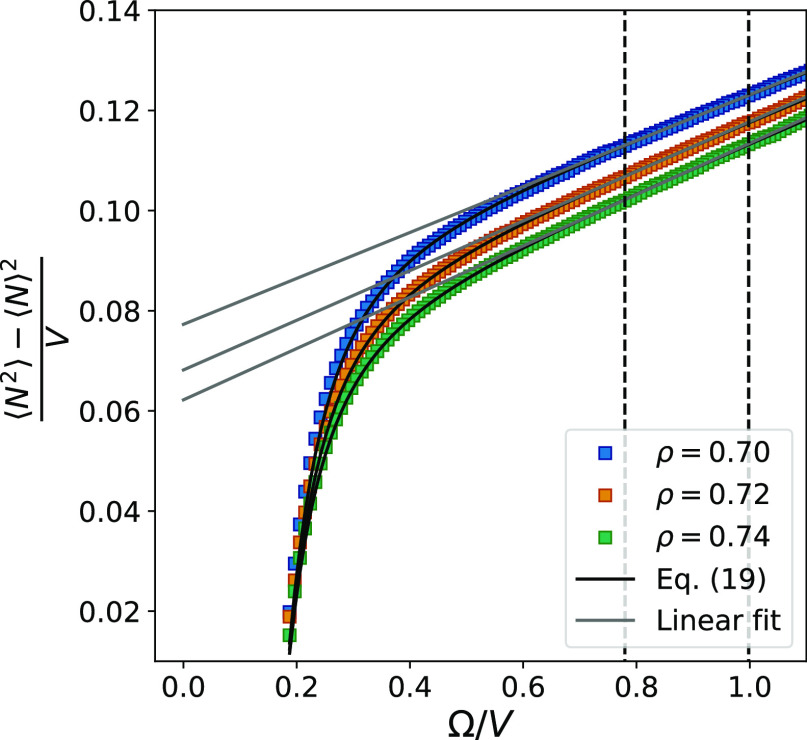
How the value of ν given by eq 18 changes with the surface-to-volume ratio of the cubic subsystem it was calculated in. For the three different curves, the density is constant, equal to that of the reservoir. The full gray line shows the result of linear fitting to the data points between the dashed lines, while the full black curve shows the result of fitting eq 19 to the data points between 0.4 < Ω/*V* < 1.0.

Figure 7 shows eq 19 fitted to all fluctuations computed in cubic subvolumes 0.4 < Ω/*V* < 1.0 and the line resulting from linear fitting between Ω/*V* = 0.78 and Ω/*V* = 1.0. The two lines show good overlap in the region between the dashed lines, which indicate the region used for the linear fit.

In conclusion, a simple analysis of *R*^2^ based on the linear fit is able provide good estimates for the location of the linear region. These estimates can be further improved by utilizing an equation that explicitly includes the effect of the finite size of the simulation box, but the effect of this additional step was marginal for the systems investigated here. The final limits identified by this method correspond to slightly smaller subvolumes than those of the previously suggested general limits.^27,35^ In the following analysis of chemical potential, we therefore analyze the regions 0.60 < Ω/*V* < 1.0 for spherical subvolumes and between 0.78 < Ω/*V* < 1.0 for cubic subvolumes.

#### Calculating Chemical Potential Differences from the SSM

Now, we will show how the distributions in the subsystems are used to calculate their chemical potential differences. Figure 5 shows the fluctuations that represent the distributions, which we apply the ODM to. Also here we use the maximum likelihood approach, which means that we apply eq 17 to the particle distributions in two subvolumes of equal size, sampled from two reservoirs at different densities. From the three densities available, Δμ is computed for the two systems with the lowest densities and for the systems with the lowest and the highest densities. Standard deviations for each value of Δμ are calculated from 20 bootstrap samples. For some of the largest subvolumes, the distributions become too far apart for the ODM analysis to converge. The 20 largest subvolumes are therefore not included in the following analysis. In the linear region for the cubic subvolumes, 0.78 < *Ω/V* < 1.0, the values calculated in both spherical and cubic subvolumes overlap. The figures presented in this section therefore only contain results from spherical subvolumes, while the corresponding figures for the cubic subvolumes are found in the Supporting Information. Relative errors for the values in the thermodynamic limit are presented for both cubic and spherical subvolumes.

**Figure 8:**
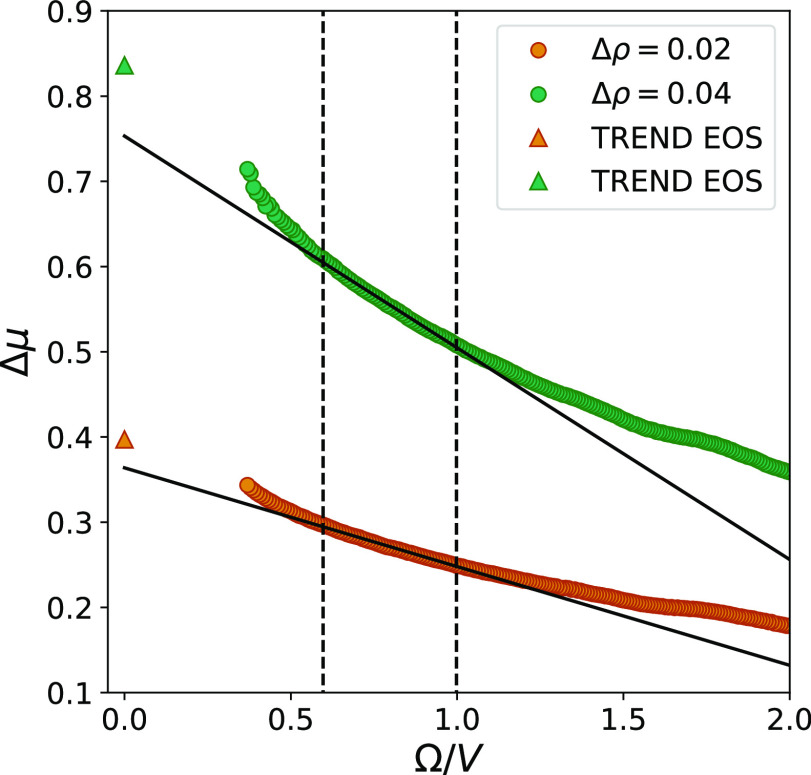
Chemical potential difference as a function of the surface-to-volume ratio. The values of Δμ were calculated by using fluctuations generated from spherical subvolumes in two separate reservoirs with different densities, combined in the maximum likelihood approach of the ODM. Error bars representing two standard deviations are included, but they are smaller than the markers.

Figure 8 shows that the values of Δμ increase with the subvolume size and that they clearly approach the correct value in the thermodynamic limit (Ω/*V* → 0). Accurate estimates of values in the thermodynamic limit are calculated from the EOS by Thol et al.^50^ The ones calculated using the Widom particle insertion method show only a 0.6% deviation from these and are therefore not included in the figure. In the following, the reported relative errors are therefore calculated with respect to the values obtained from the EOS by Thol et al.^50^

Before proceeding to investigate the accuracy of the results, one important question must be answered. How is it possible that the chemical potential inside a subvolume differs from the chemical potential in the particle reservoir to which it is connected?

The answer to this is that the reservoir and the subsystems have different types of boundaries. The reservoir has periodic boundaries, which make it behave as a macroscopic system. The subsystems, however, do not have periodic boundaries, which introduces a surface effect to their properties. We have already shown that grand canonical systems can have a size-dependent density, and we have explained that if this is the case for the subsystems sampled with the SSM, the size dependence will not appear in the densities. The sampling procedure forces all subsystems to have the same average density, which means that the size dependence instead modifies the values of the chemical potentials. Hence, the chemical potential inside subsystems will be size-dependent and different from the chemical potential of the reservoir.

The dashed lines in Figure 8 show the limits of the linear region, which means that between these lines, we find values of ν that scale linearly with the surface area-to-volume ratio. For the volumes that are too large for this region (Ω/*V* < 0.60), Δμ displays a more rapid change. While the values of ν in Figure 5 decreased at this point, the values of Δμ in Figure 8 instead increased more rapidly when approaching larger subsystems. This can be attributed to the fact that the fluctuations become too small at this point since too small fluctuations correspond to too narrow distributions, which results in higher values of Δμ.^18^

The full black lines in Figure 8 show the straight lines fitted to the data points between the dashed lines. According to eq 10, the intersection of this line corresponds to the value in the thermodynamic limit. The relative errors of Δμ^∞^ are calculated with respect to the EOS by Thol et al.^50^ and correspond to 8 and 10% (10 and 12% for the cubic subvolumes). The relative error is larger than what we can expect when using the ODM separately since it was shown to give relative errors below 2% for different types of small systems.

It is possible to improve the accuracy of the extrapolated value by plotting the data differently. When using the SSM, it has been shown that the quality of the extrapolated value depends mostly on the quality of the linear fit.^48^ Some properties have earlier been shown to have a more clear linear region if they are plotted as their inverse. This was the case for the partial enthalpies^30^ and for the thermodynamic factor.^32^ Common for both of these properties is that they are partial derivatives with respect to the number of particles. Since the chemical potential also is a partial derivative with respect to number of particles, it is interesting to see how its inverse behaves.

This idea can be further substantiated by investigating the connection between the partial derivative of chemical potential with respect to density and the particle fluctuations. The relation comes from combining eqs’s 3, 4, and 9 and is given by
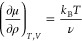
20which upon integration becomes
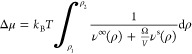
21

It can here be argued that even though the values of ν^∞^ and ν^s^ depend on density, the scaling of ν as a function of Ω/*V* remains linear. This suggests that 1/Δμ also will scale linearly as a function of Ω/*V*.

**Figure 9:**
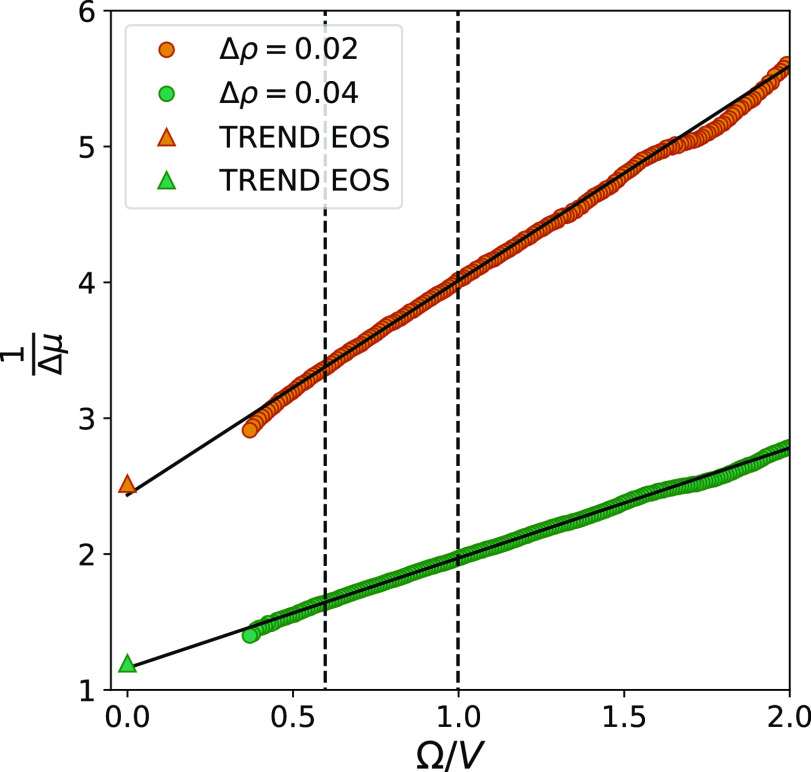
Inverse chemical potential difference as a function of the surface area-to-volume ratio. The values of Δμ were calculated by using fluctuations generated from spherical subvolumes in two separate reservoirs with different densities, combined in the maximum likelihood approach of the ODM. Error bars representing two standard deviations are included, but they are smaller than the markers.

Figure 9 shows the values of 1/Δμ as a function of the surface-to-volume ratio. This curve shows a more clear linear behavior than the ones in Figure 8. By using the same system sizes in the curve fitting, the relative errors of the extrapolated values of Δμ are reduced to 2 and 3% (3 and 4% for cubic subvolumes). This is closer to the accuracy we can expect from the ODM, and it is similar to previously reported accuracies for other properties calculated by the SSM.^29^

#### Histogram versus the Maximum Likelihood Approach of the ODM

In early applications of the ODM, histograms were used to calculate the distributions and their overlaps.^2,14,16,17^ The maximum likelihood approach by Shirts et al.^15^ is able to use the complete set of data of particle numbers instead of reducing these to histograms, which usually provides more precise and accurate results.^18^ However, the maximum likelihood approach includes an optimization step that is much more demanding with respect to both computational time and memory, compared to simply sorting the particle numbers in histograms. In this section, we will investigate if it is possible reduce time and computational cost, without loss of accuracy, by replacing the maximum likelihood approach with the original histogram version of the ODM.

**Figure 10:**
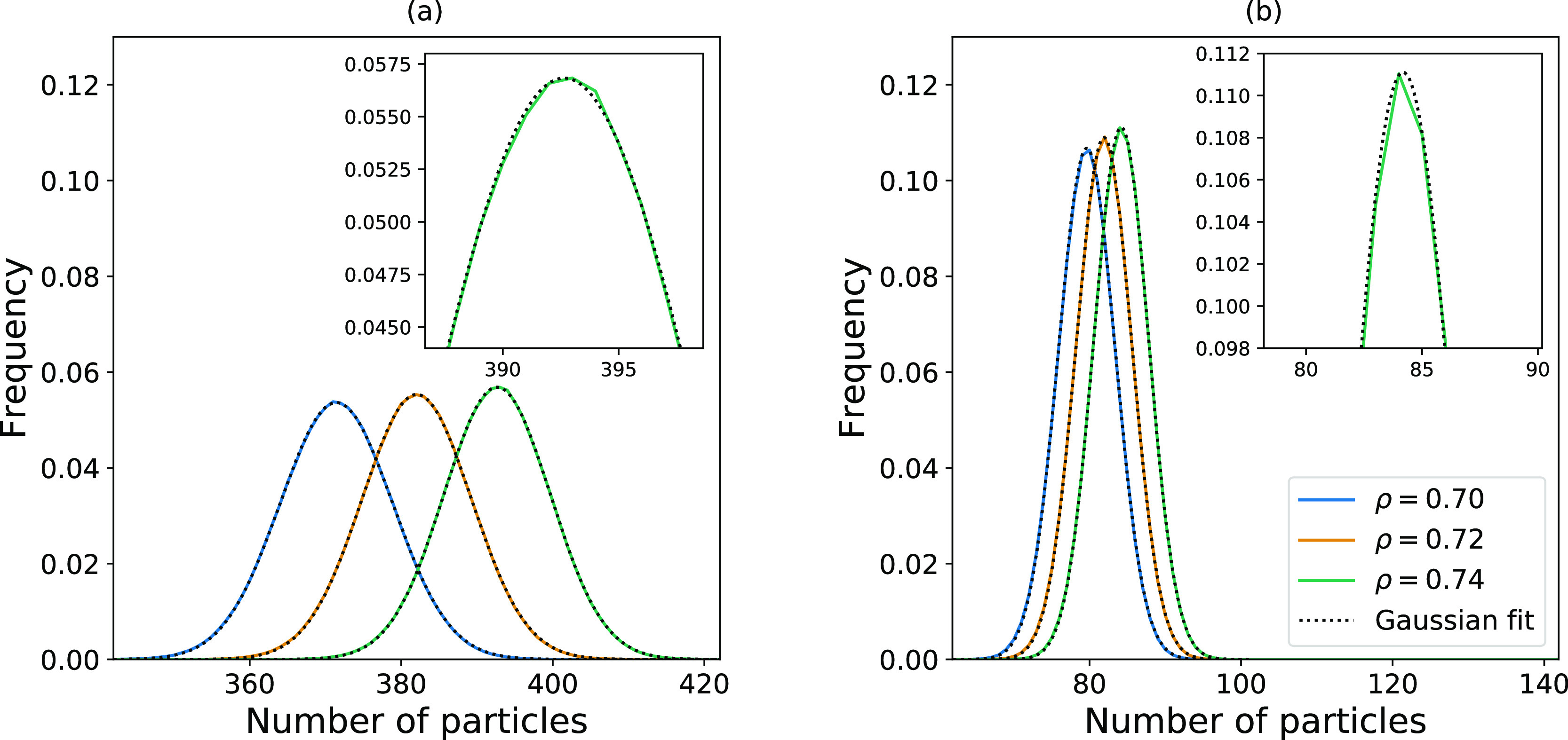
Histograms of particle distributions representing the fluctuations shown in Figure 5. (a) shows the distribution of number of particles in the largest volume in the linear region, corresponding to Ω/*V* = 0.60, while (b) shows the ones for the smallest volume in the linear region, corresponding to Ω/*V* = 1.0. The dotted lines represent the Gaussian curves fitted to the histogram data.

Another convenient aspect with histograms is that they make it possible to visually inspect the distributions. Figure 10a shows the distributions in the largest (Ω/*V* = 0.60) subsystem included in the curve fitting, and Figure 10b shows the smallest (Ω/*V* = 1.0) subsystem included in the curve fitting. The size of the *x*- and *y*-axis ranges is equal in Figure 10a,b for the total, as well as the inset figures. This makes it possible to directly compare the features of the distributions. We observe that the distributions in the smallest subsystem have poorer statistics than the distributions in the largest subsystem. For systems with such a low number of particles, the histograms will deviate more from a smooth curve, which can introduce inaccuracies in the properties calculated from the overlap. There is, however, a simple solution to this problem. As suggested by McDonald and Singer,^16^ a Gaussian curve can first be fitted to the distributions before calculating the ratio of the overlap. The fitted Gaussian curves are displayed in Figure 10, together with the histograms calculated directly from the particle numbers.

**Figure 11:**
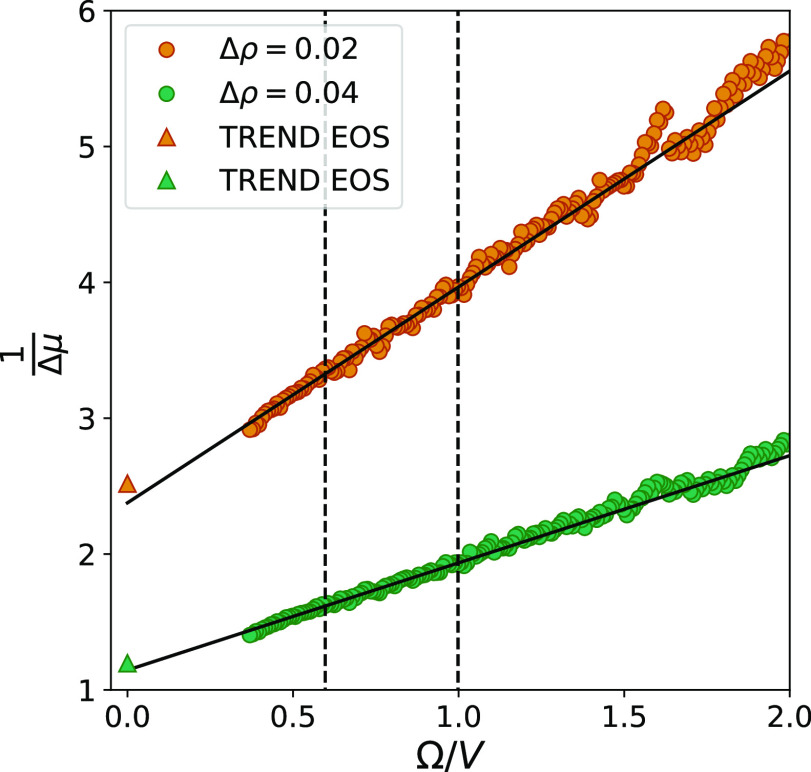
Inverse chemical potential difference as a function of the surface-to-volume ratio. The values of Δμ were calculated by using fluctuations generated from spherical subvolumes in two separate reservoirs with different densities, combined in the histogram approach of the ODM. Error bars representing two standard deviations are included, but they are smaller than the markers.

The results of applying the above described method for calculation of Δμ are shown in Figure 11. By comparing this to the results obtained from the maximum likelihood version of the ODM (Figure 9), we can see that the histogram method introduces larger variations in the values of Δμ. However, the relative error of the extrapolated value is only increased by 1%-point. This means that even though the accuracy of a single data point calculated from histograms generally is lower than the one obtained from the maximum likelihood approach, it does not have a large effect on the results from the curve fitting. As long as the uncertainties introduced by the histogram approach do not lead to a change in trend of the data, it will act as randomly distributed noise, which eventually is canceled out in the curve fitting.

Consequently, if the goal is to obtain the value of Δμ in the thermodynamic limit, the histogram approach and the maximum likelihood approach works almost equally well for the systems considered here. However, if the main interest is properties for a specific system size, the maximum likelihood method is more likely to determine this value with higher accuracy.

### Scope and Limitations

Computational methods for chemical potential differences are a field that is being continuously explored. One of the most popular methods for these investigations is the Widom particle insertion method,^12^ which is often used as a benchmark reference when new methods are presented.^39−41,47^ It is well known that the steps involving insertion or deletion of particles used in the Widom method become inefficient at higher densities. The same goes for related grand canonical particle insertion schemes such as the Gibbs ensemble MC method which often is used to investigate phase equilibria.^47^ The branch of methods that instead compute chemical potential differences from fluctuations sampled from subsystems thus have an advantage at higher densities.

In addition to the relations used to extract Δμ in this work, there also exists a connection between the differential chemical potential and the fluctuations, given by eq 20. Absolute values of chemical potential have previously been successfully extracted from this method for both one-component^38^ and multicomponent systems.^35,37^ Both the method based on the differentials of μ and the one presented here are able to provide absolute values when used in combination with another method that allows one to calculate the chemical potential at a reference state point.

One important difference between the two methods is that the method based on the differentials of μ involves numerical integration. It is therefore necessary to sample a large enough range of different densities in order to provide accurate values of μ. The method presented here is able to extract values of Δμ directly from two simulations.

Another important difference is that the method based on the differentials of μ can explicitly include the effect of the finite size of the simulation box in the scaling laws used to extract (∂μ/∂ρ)_*T*,*V*_. In contrast, the method presented here is only able to use these scaling laws to identify the subvolumes that are not affected by this feature. We have shown that explicitly including this effect returns a value of ν^∞^ with a relative error below 1%. Based on a similar analysis,^29^ this could suggest that even larger simulation box sizes are needed to further decrease the relative error computed by the method presented in this work.

Further work with the method presented here involves extension to multicomponent systems and investigation of its application to molecular fluids.

## Conclusions

We have presented a new method for computation of chemical potential differences, in both small and large systems, from molecular dynamics simulations. The new method can be seen as an extension of the SSM, which uses small subsystems embedded in a larger simulation box to calculate distributions of the number of particles. This method, which until now has been used to calculate enthalpies and the thermodynamic factor,^28^ partial molar properties,^30^ Kirkwood–Buff integrals,^32,33^ and the isothermal compressibility,^29^ has been extended to calculate chemical potential differences. This new feature was obtained by combining the SSM with an ODM. As the name suggests, the ODM uses the overlap of distributions from two different simulations to calculate thermodynamic properties. For systems with a fluctuating number of particles, one of the available properties is the chemical potential difference.

Before applying the ODM to the distributions created from the SSM, it was necessary to investigate how well the ODM performs for small systems. This was done by applying the ODM to particle distributions generated by two grand canonical MC simulations with different input values of μ, but otherwise identical parameters. Two different types of small systems were investigated, and a system with periodic boundaries was used as a reference. The values of Δμ calculated from the ODM for all of these systems had a relative error below 2%, which means that the ODM can be regarded as equally reliable for both the small and large systems investigated in this work.

The SSM generates small systems of a range of different sizes, which means that it can give insights on how an intensive property such as the chemical potential starts depending on the system size when the system becomes small enough. As a result of this, the chemical potential difference can be calculated as a function of the system size. Combined with a scaling law based on Hadwiger’s^44^ theorem, the values of Δμ can be extrapolated from the small systems to the thermodynamic limit. We also have presented tools that will be helpful in determining which system sizes should be included in this extrapolation. Compared to methods based on insertion of particles, the fluctuation-based methods have an advantage when it comes to extracting thermodynamic properties at high densities. The particular method presented here also provides an option that is independent of numerical integration.

When using the ODM, there are two options for extracting information from the overlap of the distributions. The fluctuations in number of particles can either be stored in histograms before the overlap of these is computed, or a maximum likelihood approach can be used on the complete data set. We have shown that these methods work almost equally well for determining the value of Δμ in the thermodynamic limit since they both provide values with a relative error below 4%. The maximum likelihood approach is able to determine this value with 1%-point higher accuracy. The small difference is mainly because random noise is canceled out in the curve fitting. If the main interest is one value of Δμ for a specific system size, the maximum likelihood approach will probably provide a more accurate result.

## Supporting Information Available

Chemical potential difference in cubic subvolumes (PDF)
