# Supplementary material for: Chemical Potential Differences in the Macroscopic Limit from Fluctuations in Small Systems
Source: J Chem Inf Model. 2021 Feb 10;61(2):840–55. doi: 10.1021/acs.jcim.0c01367 (PMC8023585; doi:10.1021/acs.jcim.0c01367)
Supplement: Supplementary file 1 — ci0c01367_si_001.pdf [file ci0c01367_si_001.pdf]

Supporting Information
Chemical Potential Differences in the Macroscopic Limit from Fluctuations in Small Systems

Vilde Bråten,[†] Øivind Wilhelmsen,[‡,¶] and Sondre Kvalvåg Schnell[*,§]

†*Department of Materials Science and Engineering, Norwegian University of Science and Technology, NTNU, Trondheim, NO-7491, Norway*

‡*SINTEF Energy Research, Trondheim, NO-7465, Norway*

¶*Department of Energy and Process Engineering, Norwegian University of Science and Technology, Trondheim, NO-7491, Norway*

§*Department of Materials Science and Engineering, Norwegian University of Science and Technology, NTNU, Trondheim, N-7491, Norway*

E-mail: sondre.k.schnell@ntnu.no

The Fig.'s S1, S2 and S3 represent the values of chemical potential difference, $\Delta\mu$, calculated in cubic sub-volumes.

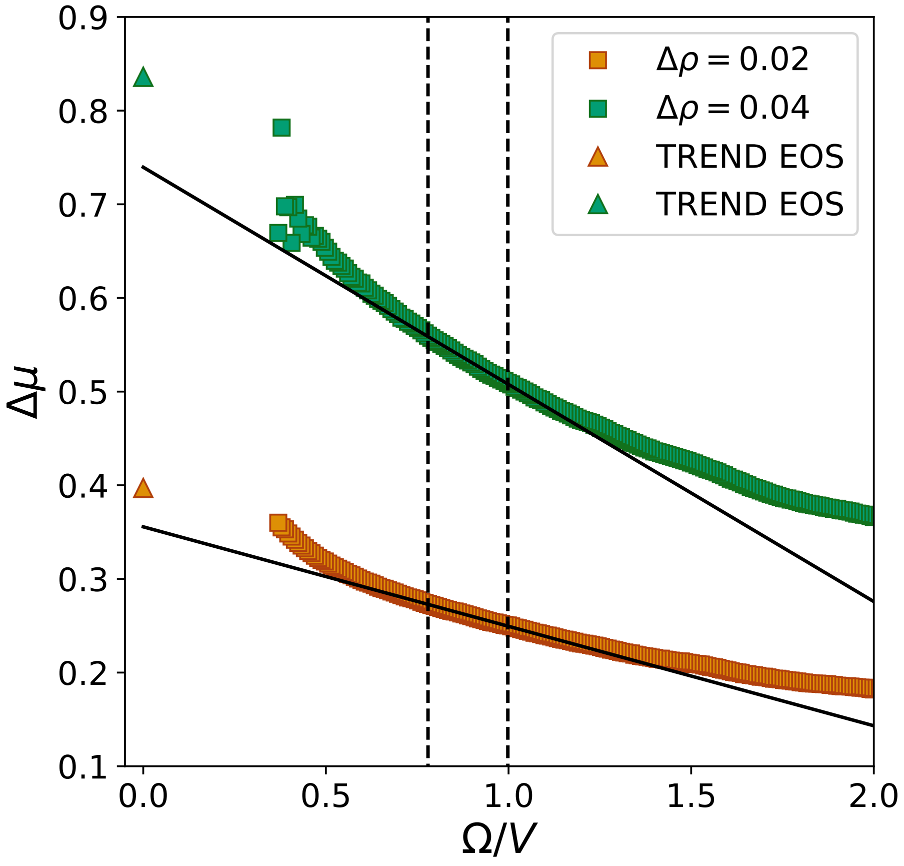

Figure S1: Chemical potential difference as a function of surface to volume ratio. The values of $\Delta\mu$ were calculated by using fluctuations generated from cubic sub-volumes in two separate reservoirs with different density, combined in the maximum likelihood approach of ODM. Error bars representing two standard deviations are included, but they are smaller than the markers.

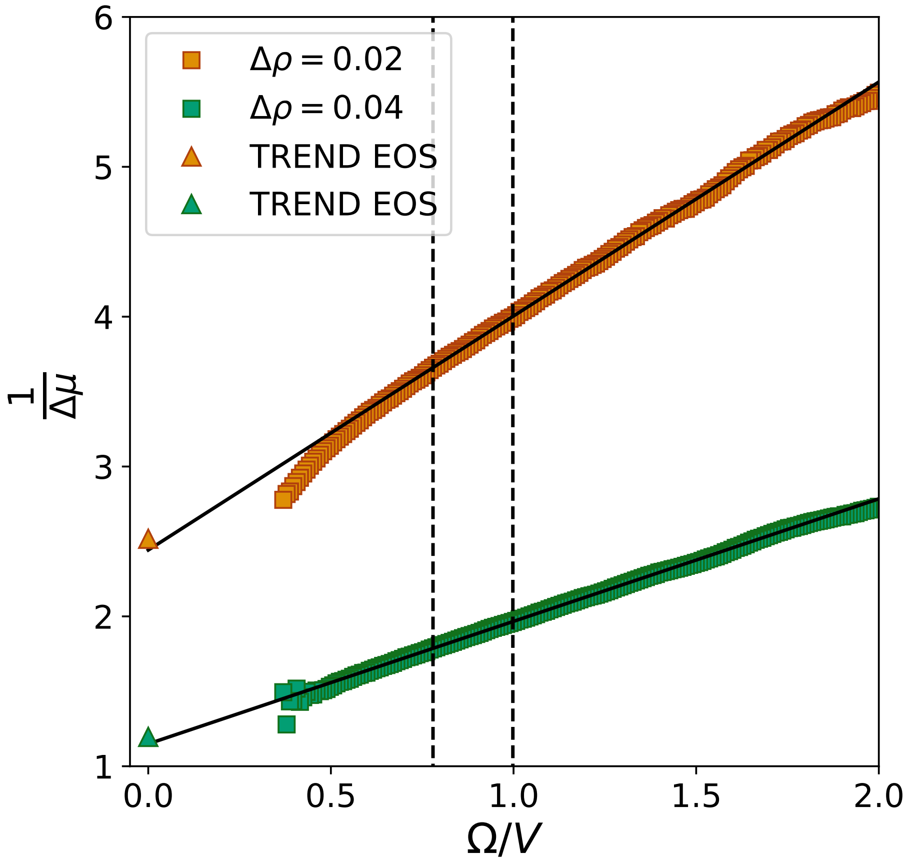

Figure S2: The inverse chemical potential difference as a function of surface area to volume ratio. The values of $\Delta\mu$ were calculated by using fluctuations generated from cubic sub-volumes in two separate reservoirs with different density, combined in the maximum likelihood approach of ODM. Error bars representing two standard deviations are included, but they are smaller than the markers.

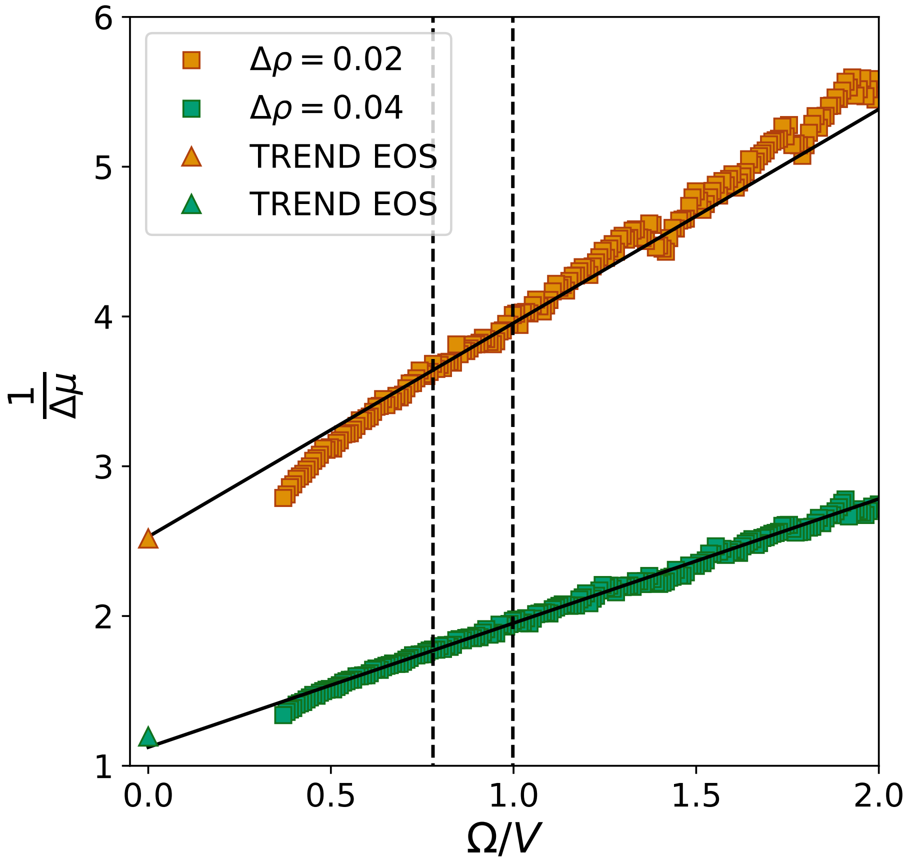

Figure S3: The inverse chemical potential difference as a function of surface to volume ratio. The values of $\Delta\mu$ were calculated by using fluctuations generated from cubic sub-volumes in two separate reservoirs with different density, combined in the histogram approach of ODM. Error bars representing two standard deviations are included, but they are smaller than the markers.
